# Comparison of diagnostic performance of GAAD, GALAD, and ASAP scores for detecting hepatocellular carcinoma in advanced liver fibrosis patients

**DOI:** 10.1515/almed-2025-0144

**Published:** 2026-01-15

**Authors:** Alberto Izquierdo-Martínez, Ángela Rojas, Ricardo Rubio-Sánchez, Inmaculada Dominguez-Pascual, Manuel Romero-Gómez, Daniel Fatela-Cantillo

**Affiliations:** Department of Clinical Biochemistry, Virgen del Rocío University Hospital, Seville, Spain; SeLiver Group at Institute of Biomedicine of Seville (IBiS), Virgen del Rocio University Hospital/CSIC/University of Seville, Seville, Spain; CIBERehd, Instituto de Salud Carlos III, Madrid, Spain; Facultad de Biología, Departamento de Fisiología, Universidad de Sevilla, Seville, Spain

**Keywords:** hepatocellular carcinoma, liver fibrosis, GAAD score, GALAD score, ASAP score, fatty liver disease

## Abstract

**Objectives:**

Alpha-fetoprotein L3 (AFP-L3 %) and protein induced by vitamin K absence-II (PIVKA-II) are used in diagnostic scores such as GAAD, GALAD, and ASAP for hepatocellular carcinoma (HCC) detection. Advanced liver fibrosis (ALF) is diagnosable by the liver fibrosis index and could be combined with these blood biomarkers for better HCC detection.

**Methods:**

This study developed an analytical framework to address the role of GAAD, GALAD, and ASAP in ALF patients as a risk score to predict HCC. By analyzing data from 21 HCC and 30 ALF patients, this analysis assessed the diagnostic accuracy of individual biomarkers, ASAP, GAAD, and GALAD.

**Results:**

GAAD slightly outperformed AFP (sensitivity: 76.2 %, specificity: 88.5 %). The combination of AFP and PIVKA-II also surpassed AFP alone. PIVKA-II and AFP-L3 showed the worst performance for identifying HCC.

**Conclusions:**

GAAD and ASAP showed comparable or slightly superior performance to AFP, suggesting potential for screening strategies that should be confirmed in larger studies.

## Introduction

Hepatocellular carcinoma (HCC) is a primary liver cancer that arises in the setting of chronic inflammation and liver damage. HCC incidence is rising, partly due to the increasing cases of metabolic dysfunction-associated steatotic liver disease (MASLD), making it the third leading cause of cancer-related deaths and the leading cause among patients with cirrhosis, partly due to poor early detection [1], 2]. MASLD is a dynamic condition, characterized by the accumulation of fat in the hepatocytes, accompanied by liver inflammation and fibrosis, where fibrosis is the strongest predictor of liver-related and overall mortality [3], 4]. However, the natural course of advanced fibrosis in MASLD remains poorly understood, highlighting the critical need for HCC early detection and further research [5].

The clinical guidelines recommend using non-invasive tools such as the nonalcoholic fatty liver disease fibrosis score (NFS) and the fibrosis-4 (FIB-4) score to estimate advanced liver fibrosis in MASLD patients [6]. In addition, imaging techniques such as transient elastography (FibroScan) and magnetic resonance elastography (MRE) are commonly used to estimate the degree of fibrosis in patients with MASLD. Early diagnosis of HCC remains a significant challenge, especially in those patients with MASLD, where the disease progression and risk factors differ from those of other liver diseases. MASLD-related HCC can develop even in the absence of advanced fibrosis or cirrhosis, complicating traditional surveillance strategies that focus primarily on patients with established cirrhosis. The American Gastroenterological Association guidelines suggest that HCC screening should be considered in all MASLD patients with cirrhosis as well as advanced liver fibrosis (e.g., stage 3 fibrosis); however, the American Association for the Study of the Liver (AASLD) does not currently recommend HCC surveillance in patients with F3 fibrosis stage [5], 7], 8]. The current screening strategy for HCC detection includes a biannual liver ultrasound (US). In addition, combining the US with serum alpha-fetoprotein (AFP) levels has also been proposed, increasing the sensitivity while decreasing specificity. There is limited data to support the use of other promising imaging modalities, such as abbreviated magnetic resonance (MR), or combined strategies with serum biomarkers [9].

Across the world, investigators try to make surveillance cost-effective tools to stratify patients at high, intermediate, and low risk for HCC and to adjust surveillance strategies accordingly. Non-invasive approaches combining serological tests with US examinations in staging liver fibrosis in MASLD are preferred. The problem is that the US has limited effectiveness due to various factors, such as difficulty in detecting early tumors, inter-operator variability, and patient-related issues, such as obesity, which can interfere with adequate liver visualization. Due to these limitations, serological markers have gained increasing attention, and their use is increasing in clinical practice [10].

Other blood tests have been claimed to improve the performance of AFP, including des-gamma carboxy-prothrombin (DCP) or protein-induced by vitamin K absence-II (PIVKA-II) and lectin-bound AFP (AFP-L3). A meta-analysis of studies focused on AFP-L3 for early HCC diagnosis showed significant heterogeneity in sensitivity, specificity, and diagnostic ratio that limits the interpretability of the results [11]. There are also proposals to combine AFP with clinical and serum biomarkers in different models. GALAD is the most accepted one, and includes AFP, AFP-L3, DCP, age, and gender [12]. The performance of the GALAD score in identifying Barcelona-Clínic Liver Cancer (BCLC) stage 0/A HCC and detecting early HCC in patients with cirrhosis was analyzed in a meta-analysis that included 19,021 patients. A high or unclear patient selection bias was observed in 16 study cohorts, primarily attributed to the retrospective case-control design. GALAD showed a sensitivity of 0.73 (95 % CI 0.66–0.79), specificity of 0.87 (95 % CI 0.81–0.91), and false positive rates ranging from 4.2 to 27.2 % for detecting early HCC. These results change only slightly when the analysis is restricted to identifying HCC within BCLC 0/A stages in the population of patients with cirrhosis, where GALAD demonstrated a pooled sensitivity, pooled specificity, and estimated AUC of 0.78 (95 % CI 0.66–0.87), 0.80 (95 % CI 0.72–0.87), and 0.86 (95 % CI 0.83–0.89), respectively. Additionally, better performance was observed in retrospective studies than in prospective studies, which also reflects the selection bias in retrospective studies. Thus, despite the promising performance of GALAD and those models that included DCP and AFP, their main limitations for implementation in clinical practice are the selection bias and the threshold values of these models for detecting early-stage HCC. Consequently, these scores should be further validated by higher-quality studies before recommending them as routine screening tools [13].

In Europe, we could identify tests to identify fibrosis like Hepamet fibrosis score (HFS), NAFLD fibrosis score (NFS), and Fibrosis-4 (FIB-4) which could be used to obtain a combination index as a first line test [14] and other commercial tests like ELF (enhanced liver fibrosis) test [6], involving hyaluronic acid (HA) and a tissue inhibitor of matrix metalloproteinase type 1 (TIMP-1) along with P3NP (aminoterminal propeptide of type 3 procollagen) used as the second step. In recent years, this two-step diagnostic algorithm that combines these factors has become widespread for stratifying patients with advanced fibrosis [13]. At the same time, other established GALAD [15] and GAAD scores have demonstrated good clinical performance in detecting early-stage HCC, and their use is also becoming widespread to improve the current surveillance strategies with single biomarkers usually having suboptimal performance.

In Asia, on the other hand, the ASAP score, which was initially verified for hepatitis B virus (HBV) of HCC, has been used more than in Europe [16], but its diagnostic performance in detecting HCC among patients with other types of chronic liver diseases remains unknown. In this context, PIVKA-II implementation has shown some challenges, such as requiring standardization of cut-off values, cost-effectiveness, and improving awareness among healthcare providers [17].

This study aimed to compare the clinical performance of the ASAP, GAAD, and GALAD algorithms, as well as single tumor biomarkers (AFP and PIVKA-II), for detecting HCC in a single-centre cohort of patients at risk of advanced fibrosis.

## Materials and methods

### Study design

All patients were recruited from the Liver and Digestive Disease Unit of the Virgen del Rocío University Hospital (Seville, Spain). The study included patients with hepatic fibrosis, with or without hepatocellular carcinoma (HCC). All patients were followed from primary care consultations using a two-step algorithm to identify fibrosis, which combined FIB-4, Hepamet Fibrosis Score (HFS), and NFS with the enhanced liver fibrosis (ELF) test. This approach involved an initial assessment using the combined fibrosis index (HFS, NFS, and FIB-4), followed by an ELF test to refine the classification of fibrosis severity.

Patients were classified into two groups: those with fibrosis (without development of HCC) and those with HCC. After that, in the liver fibrosis group, 10 cases were initially diagnosed as MASLD, 3 cases as NASH, 3 cases as alcoholic cirrhosis, and 14 showed an unknown etiology of fibrosis.

In the HCC group, 21 patients were diagnosed due to focal liver lesions detected by abdominal ultrasound and confirmed by computed tomography and/or magnetic resonance imaging, according to AASLD [7] and EASL guideline [5]. The presence of HCC was systematically excluded in all patients from the fibrosis group using the same imaging and diagnostic tests. Liver disease severity in HCC was assessed by Child-Pugh grade, and HCC staging was done using the Barcelona Clinic Liver Cancer (BCLC) system [14]. In addition, 2 patients with F3 fibrosis developed HCC during follow-up (at least 1 year) and were included in the HCC group for the analysis.

All participants signed a written informed consent before blood extraction. The study was approved by the hospital´s Ethics Committee and is in accordance with the ethical guidelines of the Declaration of Helsinki. All data (including clinical, demographic, and analytical findings) were coded and confidentially stored in a database.

### Serum sample collection and assessment

Peripheral blood samples were collected in BD Vacutainer^®^ SSTTM tubes that were centrifuged at 3,000 g for 10 min at room temperature (RT), and serum was aliquoted and frozen at −80 °C until testing. Serum levels of PIVKA-II, AFP, and AFP-L3 were measured using Elecsys^®^ assays on a cobas^®^ e 801 analyzer to calculate GAAD, GALAD and ASAP scores within less than three months of collection. The GAAD, GALAD, and ASAP scores were calculated using the following formulas.

The pre-established cut-off points to distinguish benign pathology from HCC were: AFP<20 ng/mL; AFP-L3<32.3 ng/mL; PIVKA-II<28.4 ng/mL, GALAD<2.47 (range 0–10) measured using Hepcalc calculator and data from three markers and Elecsys GAAD<2.57 (range 0–10) with use of the certified algorithm in the Navify portal with data from AFP and PIVKA-II, sex, and age.

ASAP score=−7.58 + 0.05 × age−0.58 × gender + 0.42 × Ln (AFP [ng/mL]) + 1.11 × Ln (PIVKA-II [mAU/mL]), where gender=0 for males and 1 for females [18].

The GALAD score was calculated using the following equation: GALAD score=−10.08 + 0.09 × age + 1.67 × gender + 2.34 × Ln (AFP [ng/mL]) + 0.04 × AFP-L3% + 1.33 × Ln (PIVKA-II [mAU/mL]), where gender=0 for females and 1 for males [12].

The GAAD score was calculated by using an automated system called the NAVIFY^®^ Algorithm Suite (Roche Diagnostics, Basel, Switzerland), which automatically retrieves biomarker data from the Laboratory Information System and Health Information System [19].

### Statistical analysis

Patients’ characteristics are reported as the median and standard error value for continuous variables and frequency and percentage (%) for categorical variables. The association between independent groups was tested using a chi-square test for categorical variables, when necessary, and the Mann-Whitney U test (Wilcoxon rank-sum test) for continuous variables. Associations between variables or scores and HCC risk were assessed using the Cox proportional hazards regression model. Analyses were made of the total cohort. For the classification of patients as HCC or non-HCC, pre-established cut-off points were initially applied for AFP, AFP-L3, PIVKA-II, and the GAAD, GALAD, and ASAP scores, as described above. In addition, ROC curve analysis was performed to explore cohort-specific optimal cut-off values, allowing assessment of potential improvements in diagnostic accuracy within this population. This approach was intended to validate the standard thresholds while providing exploratory insight into population-specific variations. The null hypothesis of non-association was tested; the 2-tailed probability level was set at 0.05. Statistical analysis were conducted using SPSS software 26.0.

## Results

### Baseline characteristics of the study cohort

The characteristics of the patients included in the overall cohort are summarized in Table 1. Among the 30 patients, 5 (16.7 %) had cirrhosis, while the remaining patients were classified according to the combined fibrosis index, with 21 (70.0 %) at stage F3 and 4 (13.3 %) at stage F2.

**Table 1:** Participant demographics and clinical characteristics.

	HCC (n=21)	Fibrosis (n=30)	p-Value
Age, years, median (p25–p75)	72.0 (63.5–78.5)	71.0 (62.5–77.3)	0.618
Gender, n (%) male	16 (76.2)	20 (66.7)	0.543
Risk factors, n (%)			
Obesity, n (%)	3 (14.3)	7 (23.3)	<0.001
Alcohol, n (%)	14 (66.7)	8 (26.7)	<0.001
Tobacco, n (%)	9 (42.9)	5 (16.7)	0.061
HTA, n (%)	9 (42.9)	23 (76.7)	0.020
Dyslipemia, n (%)	6 (28.6)	16 (53.3)	0.086
Diabetes, n (%)	7 (33.3)	26 (86.7)	<0.001

**Disease etiology**			

MASLD, n (%)	2 (9.5)	10 (33.3)	0.091
Steatohepatitis (MASH), n (%)	2 (9.5)	3 (10.0)	1.000
Alcoholic cirrhosis, n (%)	12 (57.1)	3 (10.0)	<0.001
VHB, n (%)	4 (19.0)	3 (10.0)	0.427
VHC, n (%)	3 (14.3)	14 (46.7)	0.019
Other	5 (23.8)	–	

**Cirrhosis status**			

Cirrhotic, n (%)	13 (61.9)	5 (16.7)	0.002
Non cirrhotic, n (%)	8 (38.1)	25 (83.3)	0.002
BCLC 0/A (early stage)	14 (66.7)	–	
BCLC B/C (advanced stage)	7 (33.3)	–	

**Fibrosis status**			

F4, n (%)	13 (61.9)	5 (16.7)	0.002
F3, n (%)	8 (38.1)	21 (70)	<0.001
F2, n (%)	–	4 (13.3)	

**Serum biomarkers and risk scores**			

AFP, ng/mL, median (p25–p75)	8.3 (2.9–128.8)	2.4 (1.2–3.8)	<0.001
AFP-L3, ng/mL, median(p25–p75)	2.0 (1.2–926.0)	1.2 (1.2–1.2)	<0.001
AFP-L3, %, median (p25–p75)	39.7 (20.7–92.6)	53.2 (33.5–97.2)	0.248
PIVKA, ng/mL, median(p25–p75)	562.2 (16.9–12001.0)	24.7 (18.2–72.6)	<0.001
GAAD score	9.4 (4.3–9.9)	2.4 (1.3–3.2)	<0.001
GALAD score	5.3 (3.9–8.7)	2.4 (1.8–4.0)	<0.001
ASAP score	4.1 (1.1–5.9)	0.04 (−0.5-1.0)	<0.001
ASAP model % (probability of HCC risk)	0.65 (0.28–0.82)	0.19 (0.15–0.27)	<0.001
Cut-off GAAD<2.57, n (%)	3 (14.3)	16 (53.5)	
Cut-off GAAD=>2.57, n (%)	18 (85.7)	11 (36.7)	
Cut off GALAD<2.47, n (%)	2 (9.5)	13 (43.3)	
Cut off GALAD=>2.47, n (%)	19 (90.5)	13 (43.3)	
Cut-off ASAP<1.89, n (%)	6 (28.5)	24 (93)	
Cut-off ASAP=>1.89, n (%)	15 (71.5)	2 (7)	

The median age in the fibrosis group was similar to the HCC group, 71 years old (62.5–77.3) compared to 72 years old (p=0.618). In both groups, the main sex was male (76.2 and 66.7 %, respectively (p=0.543)), and 83.3 % of patients had advanced liver fibrosis (F3) compared to 38.1 % in the HCC group (p=0.002), according to the fibrosis index.

In summary, those patients included in the fibrotic group showed a higher prevalence of diabetes and hypertension in comparison with HCC patients (p<0.001 and p=0.020). In contrast, alcohol consumption records showed more prevalence among the HCC group (p<0.001).

Among HCC patients, 14 (66.7 %) were classified as early-stage (BCLC 0/A) and 7 (33.3 %) as advanced-stage (BCLC B/C). Of these, 13 patients had cirrhosis and 8 were non-cirrhotic. Specifically, in the early-stage group, 3 patients were cirrhotic and 11 non-cirrhotic, while in the advanced-stage group, 4 were cirrhotic and 3 non-cirrhotic. The descriptive analysis included both fibrosis and HCC patients.

### Diagnosis accuracy of non-invasive tests: ASAP, GALAD, and GAAD

Among patients with HCC, compared with those without, AFP serum levels were significantly higher (p<0.001). In addition, AFP-L3 and PIVKA-II were also found to be increased in HCC patients (p<0.001 and p<0.001, respectively) (Table 1). The ASAP, GALAD, and GAAD scores confirmed the notable differences between the independent groups analyzed (p<0.001).

**Figure 1:**
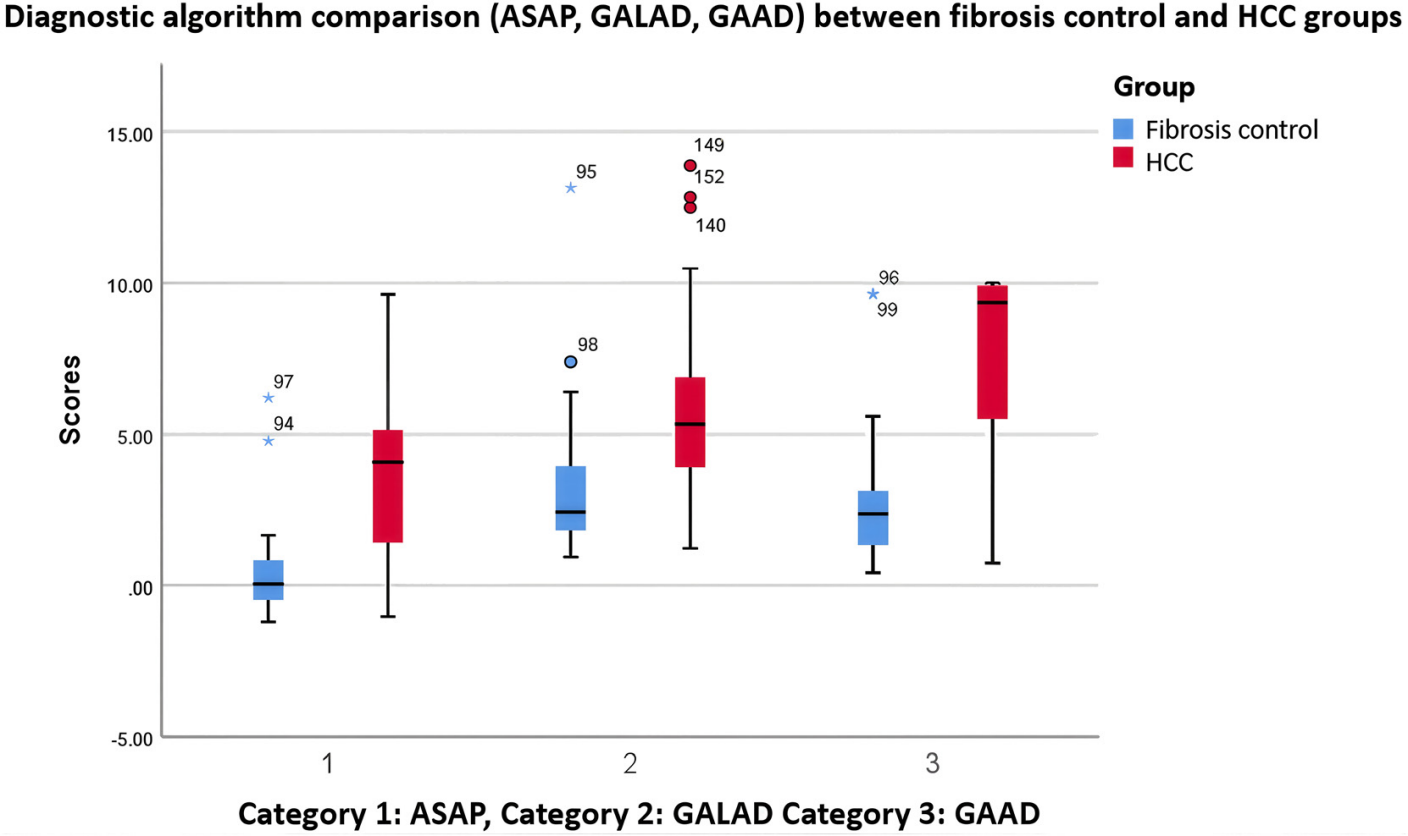
Boxplot comparing the values of diagnostic algorithms between the two groups. The analysed marker values showed significant differences between fibrosis and HCC patients, suggesting the potential diagnostic utility of these biomarkers.

Figure 1 shows a comparison of ASAP, GALAD, and GAAD scores between patients with HCC and patients with liver fibrosis. Significantly higher values are observed in the HCC group across all categories. The diagnostic performance of the GAAD, GALAD, and ASAP algorithms in identifying HCC among patients with liver fibrosis was evaluated (Table 1).

Based on the established cut-off values, the GAAD score correctly classified 85.7 % (18/21) of HCC patients, while 14.3 % (3/21) were below the threshold of 2.57. Among fibrosis patients, 53.5 % (16/30) had GAAD values below the cut-off, whereas 36.7 % (11/30) were misclassified with elevated values.

The GALAD score demonstrated a correct classification rate of 90.5 % (19/21) among HCC patients, with only 9.5 % (2/21) falling below the cut-off of 2.47. In the fibrosis group, 43.3 % (13/30) had GALAD values below the threshold, while 43.3 % (13/30) were incorrectly classified with elevated values.

Similarly, the ASAP score correctly classified 71.5 % (15/21) of HCC cases using a cut-off value of 1.89, while 28.5 % (6/21) were below the threshold. Among fibrosis patients, 93 % (24/30) were correctly classified below the cut-off, and 7 % (2/30) were falsely identified with elevated ASAP values.

The diagnostic performance of the GAAD, GALAD, and ASAP algorithms was also compared with the AFP biomarker for the HCC diagnosis. Additionally, the PIVKA-II and AFP-L3 biomarkers were included to provide a broader comparison. Sensitivity, specificity, positive predictive value (PPV), negative predictive value (NPV), and cut-off points were analyzed for each marker (Table 2).

**Table 2:** Diagnostic performance of different biomarkers and algorithms for the detection of hepatocellular carcinoma (HCC).

Markers	Sensivity	Specificity	NPV	PPV	Cut-off	Youden index
AFP, ng/mL	0.667	0.962	0.787	0.933	6.75	0.629
PIVKA-II, ng/mL	0.714	0.846	0.789	0.8125	121	0.560
AFP-L3	0.571	0.962	0.756	0.923	1.62	0.533
ASAP score	0.714	0.923	0.800	0.882	1.89	0.637
GALAD score	0.905	0.692	0.866	0.530	2.47	0.597
GAAD score	0.762	0.885	0.842	0.620	2.57	0.647

The GALAD algorithm showed the highest sensitivity (0.905), indicating a strong ability to detect HCC cases. However, it had the lowest specificity among the evaluated models (0.692), suggesting a higher rate of false positives. In contrast, AFP and AFP-L3 demonstrated the highest specificity (0.962), allowing for more accurate exclusion of negative cases, though at the expense of lower sensitivity.

The GAAD algorithm achieved a sensitivity of 0.762 and a specificity of 0.885, providing a notable balance between these two parameters. Similarly, the ASAP algorithm demonstrated comparable performance, with a sensitivity of 0.714 and specificity of 0.923, making it a robust alternative. PIVKA-II showed intermediate diagnostic performance, with a sensitivity of 0.714 and specificity of 0.846, while AFP-L3 had the lowest sensitivity (0.571) despite maintaining high specificity.

Overall diagnostic performance was further assessed using the Youden index. The GAAD algorithm had the highest Youden index (0.647), followed by ASAP (0.637) and AFP (0.533). These findings suggest that GAAD provides the best balance between sensitivity and specificity, positioning it as the most consistent model for HCC diagnosis.

**Figure 2:**
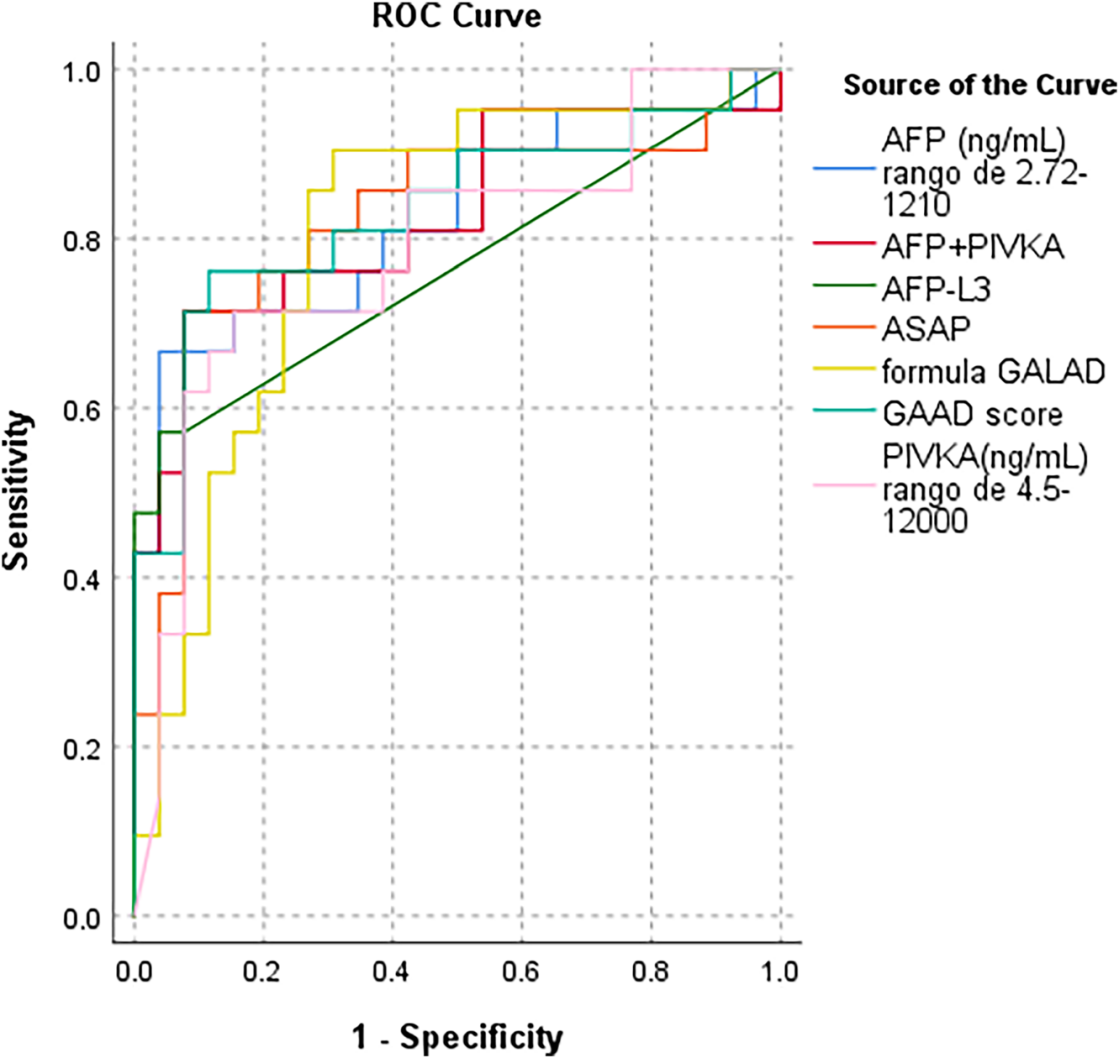
Receiver operating characteristic (ROC) curves comparing the diagnostic performance of individual biomarkers (AFP, PIVKA-II, AFP-L3) and composite models (ASAP, GAAD, GALAD) for the detection of hepatocellular carcinoma (HCC). The area under the curve (AUC) is indicated for each test. Higher AUC values reflect better discriminative ability.

Receiver operating characteristic (ROC) curve analysis was performed for all biomarkers (Figure 2) to evaluate their discriminative ability between fibrosis and HCC. Table 3 presents the area under the curve (AUC) values for each parameter.

**Table 3:** Area under the receiver operating characteristic (ROC) curve (AUC) for individual biomarkers and composite algorithms in the detection of hepatocellular carcinoma (HCC). Higher AUC values indicate better diagnostic performance.

Test result variable (s)	Area	Asymptotic 95 % confidence interval		Cut-off	Sensitivity	Specificity
		Lower bound	Upper bound			
AFP, ng/mL, range of 2.72–1,210	0.824	0.697	0.952	6.75	0.667	0.962
PIVKA-II, ng/mL, range of 4.5–12,000	0.787	0.649	0.924	121	0.714	0.846
AFP-L3, ng/mL	0.766	0.619	0.912	1.62	0.571	0.962
ASAP score	0.824	0.693	0.955	1.89	0.714	0.923
GALAD score	0.806	0.676	0.936	3.64	0.905	0.692
GAAD score	0.833	0.708	0.959	5.24	0.762	0.885

The results showed that GAAD had the best diagnostic performance with an AUC of 0.833, followed by the AFP+PIVKA-II combination (0.826), and both ASAP and AFP, each with an AUC of 0.824. GALAD achieved an AUC of 0.806, while PIVKA-II and AFP-L3 had AUCs of 0.787 and 0.766, respectively. These results highlight variations in diagnostic accuracy, with GAAD demonstrating the highest performance in differentiating between fibrosis and HCC.

When comparing the diagnostic performance of the models between patients with advanced fibrosis and those with cirrhosis, a decline in accuracy was observed for all algorithms. GALAD showed the greatest reduction, achieving an AUC of 0.603, whereas GAAD and ASAP maintained higher AUCs of 0.708 and 0.722, respectively.

## Discussion

The results obtained in this cohort highlight the clinical relevance of the GAAD, GALAD, and ASAP diagnostic models for the HCC identification in patients with advanced liver fibrosis. In our population, composed mainly of patients with F3 fibrosis (86.6 %) and non-cirrhotic etiology (83.3 %), we observed a clear clinical and demographic differentiation between the fibrosis and HCC groups. While patients with fibrosis more frequently presented with metabolic comorbidities such as diabetes and hypertension, those in the HCC group showed a male predominance (76.2 %), older age (median of 72 years), and higher alcohol consumption, reflecting the heterogeneity of risk factors involved in tumor development.

It should be considered that the underlying etiology of liver disease may influence the diagnostic utility of these biomarkers. For example, AFP tends to be elevated in viral hepatitis and cirrhosis [20], whereas PIVKA-II has shown superior performance in HBV-related HCC [21], particularly in Asian populations. In MASLD, available data are more heterogeneous and remain limited. Because our cohort included patients with diverse etiologies (MASLD, alcohol-related, viral hepatitis, and unknown), we were unable to perform stratified sub-analyses, which represents a relevant limitation. Previous studies have reported meaningful differences in the diagnostic accuracy of AFP, PIVKA-II, and composite scores such as GAAD, GALAD, and ASAP depending on the underlying etiology [18], [22], [23], [24].

From a diagnostic standpoint, the GAAD model was the best-performing algorithm overall, achieving a sensitivity of 76.2 %, a specificity of 88.5 %, and a Youden index of 0.647. These results position GAAD as the most balanced tool for discriminating between advanced fibrosis and HCC, slightly outperforming ASAP (sensitivity of 71.4 %, specificity of 92.3 %, and a Youden index of 0.637) and clearly surpassing GALAD, whose higher sensitivity (90.5 %) was accompanied by lower specificity (69.2 %), increasing the likelihood of false positives. This trend was consistent in the area under the curve (AUC) analysis, where GAAD reached the highest value (0.833), even outperforming biomarker combinations such as AFP+PIVKA-II (0.826). Although GALAD showed good performance (AUC=0.806), it did not reach the level of precision expected for an ideal screening algorithm.

Interestingly, AFP alone achieved a higher AUC (0.824) than AFP-L3 (0.766) in discriminating fibrosis from HCC. This may reflect the characteristics of our cohort: AFP can be elevated in advanced fibrosis before full tumor development [25], while AFP-L3, being more specific, is more sensitive in HCC associated with viral hepatitis and in early-stage tumors [26]. The predominance of MASLD and alcohol-related etiologies, along with the small sample size, may have further limited the discriminatory capacity of AFP-L3.

Importantly, although the AUC values of the composite scores and the ASAP model did not significantly exceed that of AFP alone, it is important to note that AFP levels remained within the normal range in many patients, whereas PIVKA-II was more frequently elevated above the diagnostic threshold. This pattern suggests that the performance of the composite scores is largely driven by PIVKA-II, which likely contributes more strongly to their discriminatory capacity than AFP. These findings highlight the relevance of PIVKA-II as a key biomarker, particularly when integrated with other clinical variables and markers to enhance diagnostic accuracy in patients with advanced fibrosis and increased risk of HCC.

Another important finding was the diagnostic performance comparison between the fibrosis and cirrhosis groups. When applying the models to differentiate between HCC and cirrhosis, all algorithms showed a decline in diagnostic accuracy. Specifically, GALAD was the most affected (AUC=0.603), suggesting a reduced capacity to discriminate tumor lesions in the context of established cirrhosis. This is clinically significant, given that cirrhotic patients represent a high-risk population in which early HCC diagnosis is particularly critical. In contrast, GAAD and ASAP maintained acceptable performance (AUCs of 0.708 and 0.722, respectively), suggesting they may still be useful tools even in this subpopulation.

The high false positive rate observed with GALAD in patients with advanced fibrosis (43.3 % misclassified above the threshold) reinforces the need for caution when interpreting its results, especially in low-risk settings or in the absence of supportive clinical data, despite GALAD identifying a slightly higher number of HCC cases. This illustrates that GALAD prioritizes sensitivity at the expense of specificity. On the other hand, both GAAD and ASAP showed lower rates of false positives and false negatives, making them potentially more useful tools for risk stratification and the planning of follow-up strategies in patients with MASLD and fibrosis. It should be emphasized that standard cut-off values were used for patient classification, while ROC-derived thresholds were explored only to assess possible cohort-specific variations.

Taken together, these data underscore the importance of implementing a multimodal approach to HCC detection, integrating biomarker-based algorithms (such as GAAD or ASAP) clinical tools, imaging techniques, and longitudinal follow-up. Risk-based stratification, particularly in patients with non-cirrhotic advanced fibrosis, represents an emerging field that could greatly benefit from these strategies.

In this context, our findings open the possibility of designing a stepwise clinical screening protocol in which, following an initial assessment using combined fibrosis index (such as FIB-4, NFS, or Hepamet), patients at high risk of advanced fibrosis could undergo a second evaluation using more specific algorithms such as GAAD or ASAP. These models, by demonstrating a good balance between sensitivity and specificity, could be incorporated as a triage tool to optimize referrals to hepatology specialists, avoiding both underdiagnosis and unnecessary overload of the healthcare system.

This approach would allow for better stratification of at-risk populations, prioritizing those who truly require closer surveillance or advanced imaging studies. Moreover, its implementation would contribute to a more rational use of healthcare resources, facilitating the early identification of HCC in potentially curable stages, especially in patients with non-cirrhotic etiology who are often excluded from conventional surveillance programs. The integration of these algorithms into clinical practice, through digital tools or automated protocols, could represent a significant shift in the secondary prevention of HCC.

However, the primary limitation of this study is the small sample size, which significantly restricts the strength and generalizability of the conclusions. The number of patients included is insufficient to draw robust inferences about the diagnostic accuracy of these models, and the reported sensitivity, specificity, and AUC values should be interpreted with caution given the likely wide confidence intervals. Therefore, the observed trends should be viewed as preliminary.

While GAAD and ASAP showed promising performance in this limited cohort, their potential utility for HCC screening or risk stratification must be validated in larger, prospective, and externally confirmed studies before clinical implementation can be recommended.

Future research involving broader, multi-center cohorts and diverse etiologies of chronic liver disease will be essential to confirm these findings and refine the diagnostic thresholds for these algorithms.

## References

[1] Sung H, Ferlay J, Siegel RL, Laversanne M, Soerjomataram I, Jemal A (2021). Global cancer statistics 2020: GLOBOCAN estimates of incidence and mortality worldwide for 36 cancers in 185 countries. CA Cancer J Clin.

[2] Eslam M, Newsome PN, Sarin SK, Anstee QM, Targher G, Romero-Gomez M (2020). A new definition for metabolic dysfunction-associated fatty liver disease: an international expert consensus statement. J Hepatol.

[3] Vilar-Gomez E, Calzadilla-Bertot L, Wai-Sun V, Castellanos M, Aller-de la Fuente R, Metwally M (2018). Fibrosis severity as a determinant of cause-specific mortality in patients with advanced nonalcoholic fatty liver disease: a multi-national cohort study. Gastroenterology.

[4] Roehlen N, Crouchet E, Baumert TF (2020). Liver fibrosis: mechanistic concepts and therapeutic perspectives. Cells.

[5] Forner A, Llovet JM, Mazzaferro V, Piscaglia F, Raoul JL, European Association for the Study of the Liver (2018). EASL clinical practice guidelines: Management of hepatocellular carcinoma. J Hepatol.

[6] Kjaergaard M, Lindvig KP, Thorhauge KH, Andersen P, Hansen JK, Kastrup N (2023). Using the ELF test, FIB-4 and NAFLD fibrosis score to screen the population for liver disease. J Hepatol.

[7] Singal AG, Llovet JM, Yarchoan M, Mehta N, Heimbach JK, Dawson LA (2023). AASLD practice guidance on prevention, diagnosis, and treatment of hepatocellular carcinoma. Hepatol Baltim Md.

[8] Loomba R, Lim JK, Patton H, El-Serag HB (2020). AGA clinical practice update on screening and surveillance for hepatocellular carcinoma in patients with nonalcoholic fatty liver disease: expert review. Gastroenterology.

[9] Tzartzeva K, Obi J, Rich NE, Parikh ND, Marrero JA, Yopp A (2018). Surveillance imaging and alpha fetoprotein for early detection of hepatocellular carcinoma in patients with cirrhosis: a meta-analysis. Gastroenterology.

[10] Shahini E, Pasculli G, Solimando AG, Tiribelli C, Cozzolongo R, Giannelli G (2023). Updating the clinical application of blood biomarkers and their algorithms in the diagnosis and surveillance of hepatocellular carcinoma: a critical review. Int J Mol Sci.

[11] Wang X, Zhang Y, Yang N, He H, Tao X, Kou C (2020). Evaluation of the combined application of AFP, AFP-L3 %, and DCP for hepatocellular carcinoma diagnosis: a meta-analysis. Biomed Res Int.

[12] Cagnin S, Donghia R, Martini A, Pesole PL, Coletta S, Shahini E (2023). Galad score as a prognostic marker for patients with hepatocellular carcinoma. Int J Mol Sci.

[13] Guan MC, Zhang SY, Li N, Ding Q, Fu TT, Zhang GX (2023). The performance of GALAD score for diagnosing hepatocellular carcinoma in patients with chronic liver diseases: a systematic review and meta-analysis. J Clin Med.

[14] Ampuero J, Aller R, Gallego-Durán R, Crespo J, Calleja JL, García-Monzón C (2020). Significant fibrosis predicts new-onset diabetes mellitus and arterial hypertension in patients with NASH. J Hepatol.

[15] Piratvisuth T, Tanwandee T, Thongsawat S, Sukeepaisarnjaroen W, Esteban JI, Bes M (2022). Multimarker panels for detection of early stage hepatocellular carcinoma: a prospective, multicenter, case-control study. Hepatol Commun.

[16] Yang T, Xing H, Wang G, Liu M, Yan C, Li H (2019). A novel online calculator based on serum biomarkers to detect hepatocellular carcinoma among patients with hepatitis B. Clin Chem.

[17] Kim DY, Toan BN, Tan CK, Hasan I, Setiawan L, Yu ML (2023). Utility of combining PIVKA-II and AFP in the surveillance and monitoring of hepatocellular carcinoma in the Asia-Pacific region. Clin Mol Hepatol.

[18] Liu SY, Li C, Sun LY, Guan MC, Gu LH, Yin DX (2022). ASAP score versus GALAD score for detection of hepatitis C-related hepatocellular carcinoma: a multicenter case-control analysis. Front Oncol.

[19] Piratvisuth T, Hou J, Tanwandee T, Berg T, Vogel A, Trojan J (2023). Development and clinical validation of a novel algorithmic score (GAAD) for detecting HCC in prospective cohort studies. Hepatol Commun.

[20] Notarpaolo A, Layese R, Magistri P, Gambato M, Colledan M, Magini G (2017). Validation of the AFP model as a predictor of HCC recurrence in patients with viral hepatitis-related cirrhosis who had received a liver transplant for HCC. J Hepatol.

[21] Si YQ, Wang XQ, Fan G, Wang CY, Zheng YW, Song X (2020). Value of AFP and PIVKA-II in diagnosis of HBV-related hepatocellular carcinoma and prediction of vascular invasion and tumor differentiation. Infect Agents Cancer.

[22] Chan HLY, Hu Y, Malinowsky K, Madin K, Kroeniger K, Hou J (2024). Prospective appraisal of clinical diagnostic algorithms for hepatocellular carcinoma surveillance in Chinese patients with chronic hepatitis B infection. Sci Rep.

[23] Liu S, Sun L, Yao L, Zhu H, Diao Y, Wang M (2022). Diagnostic performance of AFP, AFP-L3, or PIVKA-II for hepatitis C virus-associated hepatocellular carcinoma: a multicenter analysis. J Clin Med.

[24] Singal AG, Tayob N, Mehta A, Marrero JA, El‐Serag H, Jin Q (2022). GALAD demonstrates high sensitivity for HCC surveillance in a cohort of patients with cirrhosis. Hepatology.

[25] Durazo F, Blatt LM, Corey W, Lin J, Han S, Saab S (2008). Des‐γ‐carboxyprothrombin, α‐fetoprotein and AFP‐L3 in patients with chronic hepatitis, cirrhosis and hepatocellular carcinoma. J Gastroenterol Hepatol.

[26] Liu S, Sun L-Y, Yao L, Zhu H, Diao Y, Wang M (2022). Diagnostic performance of AFP, AFP-L3, or PIVKA-II for hepatitis C virus-associated hepatocellular carcinoma: a multicenter analysis. Stomatology.

